# A Review of Advanced Hydrogel Applications for Tissue Engineering and Drug Delivery Systems as Biomaterials

**DOI:** 10.3390/gels10110693

**Published:** 2024-10-25

**Authors:** Hoon Choi, Wan-Sun Choi, Jin-Oh Jeong

**Affiliations:** 1 Department of Anesthesiology and Pain Medicine, Seoul St. Mary’s Hospital, College of Medicine, The Catholic University of Korea, Seoul 06591, Republic of Korea; hoonie83@catholic.ac.kr; 2 Department of Orthopaedic Surgery, Ajou University School of Medicine, Suwon 16499, Republic of Korea; cws517@hanmail.net; 3 Wake Forest Institute for Regenerative Medicine (WFIRM), Wake Forest School of Medicine, Winston-Salem, NC 27157, USA

**Keywords:** hydrogel, biomaterials, tissue engineering, regeneration, drug delivery

## Abstract

Hydrogels are known for their high water retention capacity and biocompatibility and have become essential materials in tissue engineering and drug delivery systems. This review explores recent advancements in hydrogel technology, focusing on innovative types such as self-healing, tough, smart, and hybrid hydrogels, each engineered to overcome the limitations of conventional hydrogels. Self-healing hydrogels can autonomously repair structural damage, making them well-suited for applications in dynamic biomedical environments. Tough hydrogels are designed with enhanced mechanical properties, enabling their use in load-bearing applications such as cartilage regeneration. Smart hydrogels respond to external stimuli, including changes in pH, temperature, and electromagnetic fields, making them ideal for controlled drug release tailored to specific medical needs. Hybrid hydrogels, made from both natural and synthetic polymers, combine bioactivity and mechanical resilience, which is particularly valuable in engineering complex tissues. Despite these innovations, challenges such as optimizing biocompatibility, adjusting degradation rates, and scaling up production remain. This review provides an in-depth analysis of these emerging hydrogel technologies, highlighting their transformative potential in both tissue engineering and drug delivery while outlining future directions for their development in biomedical applications.

## 1. Introduction

Hydrogels have evolved significantly from their origins as simple water-absorbent materials to sophisticated platforms in biomedical engineering, first emerging in the 1950s [1,2,3]. Initially, hydrogels were utilized in products such as contact lenses and basic wound dressings, where their biocompatibility and ability to maintain a moist environment were beneficial [4,5]. Their primary value lay in their capacity to retain substantial amounts of water [6]. However, as biomedical science advanced, it became evident that earlier hydrogels had several limitations [7]. These included low mechanical strength, lack of precise control over drug release, and an inability to replicate the complex biological environments of human tissues, which limited their broader application in more demanding medical contexts [7,8,9].

Recent advancements in polymer chemistry, materials science, and bioengineering have driven the development of hydrogels with enhanced properties [10,11]. The mechanical properties of hydrogels are influenced by a variety of factors beyond fabrication techniques alone. Key variables such as crosslinking density, monomer molecular weight, concentration, and monomer composition play critical roles in determining the physical characteristics and functional performance of hydrogels. First, crosslinking density significantly affects the network structure of hydrogels, directly influencing their mechanical strength and deformability [12]. A higher crosslinking density results in increased rigidity, while lower crosslinking density allows for greater flexibility and softness. This tunability makes it possible to tailor the hydrogel’s mechanical properties for specific applications. Second, the molecular weight of the monomer is another important factor. Higher molecular weight monomers contribute to the formation of longer polymer chains, leading to stronger and more resilient networks, which in turn enhance the durability and resistance to deformation of the hydrogel [13]. Third, the concentration of monomers is critical in controlling the physical properties of hydrogels. Higher monomer concentrations produce denser networks, which improve mechanical strength and viscoelasticity [14]. Conversely, lower concentrations result in more flexible hydrogels with greater deformability, making them suitable for different types of biological applications. Finally, the chemical composition of the monomers used in hydrogel synthesis plays a fundamental role in determining the physical characteristics of the material. Specific monomers can enhance the mechanical properties of hydrogels through hydrophobic interactions or ionic bonding, while others can be chosen to provide desired functionality in particular physiological environments. Overall, the mechanical properties of hydrogels are the result of a complex interplay between these variables. By understanding and manipulating these factors, hydrogels can be engineered to meet the specific requirements of various biomedical applications. This expanded discussion offers a more comprehensive understanding of hydrogel properties and their potential for customization in tissue engineering and drug delivery. Modern hydrogels can emulate the extracellular matrix (ECM) of biological tissues, respond to specific environmental cues, deliver therapeutic agents with precision, and even autonomously repair themselves when damaged [8,15]. These improvements have significantly expanded the use of hydrogels in biomedical applications, establishing them as essential materials in areas such as tissue engineering, regenerative medicine, and precision drug delivery systems [16,17,18].

**Figure 1:**
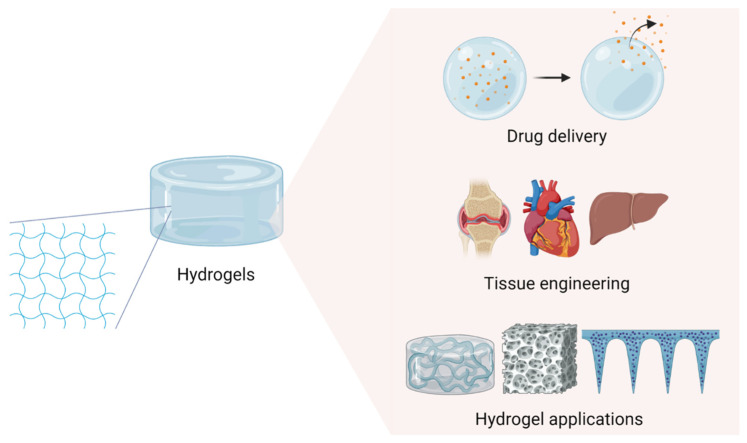
Applications of hydrogel in various fields.

This review paper offers a novel perspective by systematically analyzing recent advancements in hydrogel technology, specifically focusing on innovative types such as self-healing, tough, smart, and hybrid hydrogels (Table 1). These advanced hydrogels address the limitations of conventional hydrogels and present significant opportunities for biomedical applications. Self-healing hydrogels, with their ability to autonomously repair structural damage, are particularly promising for dynamic biomedical environments, while tough hydrogels, with enhanced mechanical properties, are well-suited for load-bearing applications such as cartilage regeneration. Smart hydrogels, which respond to external stimuli, offer tailored solutions for controlled drug release based on specific medical conditions. Hybrid hydrogels, composed of both natural and synthetic polymers, combine bioactivity and mechanical strength, offering a robust platform for complex tissue engineering. By highlighting these emerging hydrogel technologies, this review emphasizes their transformative potential and outlines key challenges and future directions, contributing to the ongoing development of hydrogel applications in tissue engineering and drug delivery systems (Figure 1).

## 2. Type of Advanced Hydrogels

### 2.1. Self-Healing Hydrogels

Self-healing hydrogels exemplify the advancements in materials science that address the limitations of earlier biomaterials [26,27]. These hydrogels are engineered to autonomously repair structural damage, thereby extending their functional lifespan and enhancing reliability in diverse conditions. The self-repairing capabilities of these hydrogels are primarily due to the incorporation of dynamic covalent bonds or non-covalent interactions within the polymer matrix, which enable the material to restore its original structure after mechanical stress or environmental changes [28,29,30]. Dynamic covalent bonds, such as Schiff base linkages, disulfide bonds, and boronate ester bonds, are crucial for self-healing hydrogels [31,32]. For instance, Schiff base linkages form imine bonds between an aldehyde and an amine, which can readily form and break under physiological conditions, making them suitable for biomedical applications like wound healing and tissue scaffolding [33,34]. Disulfide bonds, formed between two thiol groups, respond to redox conditions, allowing the hydrogel to repair itself in environments with oxidative stress. In addition to dynamic covalent bonds, non-covalent interactions, such as hydrogen bonding, ionic interactions, and host-guest chemistry, are essential for designing self-healing hydrogels [35]. Although these interactions are generally weaker than covalent bonds, they offer high reversibility, which is critical for repeated self-repair cycles [27,31]. For example, hydrogen bonds can create hydrogels that respond to temperature or pH changes, making them suitable for applications involving varying environmental conditions [27,34,36]. Host-guest chemistry involves the reversible binding of host and guest molecules, allowing the creation of hydrogels that respond to specific biological signals, enhancing their versatility in biomedical applications [26,37]. The potential applications of self-healing hydrogels are extensive. In tissue engineering, these materials can be used to fabricate scaffolds that maintain their structural integrity over long periods, even under mechanical stress [38,39,40]. This is particularly beneficial in cartilage repair, where the scaffold must endure mechanical forces while supporting tissue regeneration [26,27,41]. In drug delivery, self-healing hydrogels can facilitate the sustained release of therapeutic agents even after mechanical disruption [27,42,43]. For example, a self-healing hydrogel could continuously release medication over time, adapting to the changing conditions of the wound environment in treating chronic wounds [6,44,45]. Despite their promising features, self-healing hydrogels face several challenges. One primary concern is balancing mechanical strength with self-repair capabilities [46,47]. Incorporating dynamic bonds may weaken the material, making it less suitable for load-bearing applications [48,49]. To mitigate this, hybrid materials combining self-healing properties with reinforcing agents like nanoparticles and nanofibers are being developed to enhance mechanical strength without sacrificing healing potential [50,51,52]. Additionally, ensuring the biocompatibility and safety of self-healing hydrogels for long-term medical use remains a critical challenge [27,53,54]. Reactive chemical groups necessary for self-healing could potentially interact with surrounding tissues or degrade into harmful byproducts, necessitating careful optimization of the chemical composition of hydrogels [55].

Self-healing hydrogels have garnered significant attention in tissue engineering and drug delivery due to their ability to autonomously repair structural damage. These hydrogels are typically fabricated through either covalent or non-covalent interactions (e.g., hydrogen bonding, ionic interactions, hydrophobic interactions), which create a dynamic network capable of restoring itself after physical or chemical damage. The fabrication process can be optimized by using natural polymers, such as polysaccharide-based hydrogels, to enhance biocompatibility and biodegradability, or synthetic polymers to improve mechanical properties and fine-tune the hydrogel performance. The self-healing mechanism is primarily based on reversible interactions within the hydrogel network. For instance, multiple hydrogen bonding, ionic interactions, and dynamic covalent bonds can enable the hydrogel to recover its structure after transient damage. Furthermore, the healing efficiency and rate can be controlled by external environmental factors, such as pH, temperature, or ionic strength, allowing for the modulation of the hydrogel behavior to suit specific biomedical applications. The mechanical properties, physical stability, and drug release profiles of self-healing hydrogels can also be finely tuned. By adjusting the crosslinking density, polymer chain length, or the strength of hydrogen bonds within the network, it is possible to control the hydrogel mechanical strength and healing capacity. This allows for the design of hydrogels tailored to meet the specific requirements of various medical environments, making them versatile tools in regenerative medicine. While the sustained drug release characteristics of self-healing hydrogels are indeed a key advantage, they offer several other significant benefits. Self-healing hydrogels are particularly useful in load-bearing tissues, such as cartilage, where their ability to recover from structural damage is essential. Moreover, the physical properties of these hydrogels, such as softness and viscoelasticity, can be modulated to support both tissue regeneration and customized drug delivery systems. This ensures that the hydrogel remains functional even after damage, maintaining its therapeutic efficacy throughout the healing process. Given these properties, self-healing hydrogels hold great potential in a variety of biomedical applications, including tissue engineering, wound healing, and sustainable drug delivery systems. Their ability to rapidly recover from damage and provide durability under repetitive stress makes them particularly suitable for dynamic biological environments. As a result, they represent a promising material for future innovations in regenerative medicine and drug delivery platforms. Future research on self-healing hydrogels will likely focus on improving mechanical properties, optimizing healing capabilities, and expanding their use in biomedical fields. As our understanding of the underlying mechanisms of self-healing continues to grow, these materials are expected to become increasingly sophisticated, offering novel solutions to complex biomedical engineering challenges.

### 2.2. Tough Hydrogels

Tough hydrogels have emerged as a solution to the inherent mechanical fragility of conventional hydrogels [56,57,58]. While tough hydrogels are advantageous in many biomedical applications due to their biocompatibility and high water content, they often lack the mechanical strength required for load-bearing uses such as cartilage repair or the construction of artificial ligaments and tendons [59,60,61]. To address this issue, various design strategies have been employed to enhance the resistance of tough hydrogels to mechanical stress without compromising their flexibility or biocompatibility [62,63,64]. One of the most effective strategies for toughening hydrogels is the incorporation of a double network (DN) structure. This approach involves creating two interpenetrating polymer networks within the hydrogel matrix [65]. The first network typically consists of a rigid, brittle polymer that provides high mechanical strength, while the second network is composed of a more flexible, ductile polymer that absorbs energy and prevents failure [66,67]. The synergy between these two networks allows the hydrogel to withstand significant deformation and stress, making it ideal for applications where mechanical resilience is crucial [68]. The concept of DN hydrogels was first introduced by Gong and colleagues in 2003 [69]. They demonstrated that a DN hydrogel composed of poly(2-acrylamido-2-methylpropanesulfonic acid) (PAMPS) and polyacrylamide (PAM) exhibited extraordinary toughness compared to conventional hydrogels. This discovery has led to the widespread adoption of DN hydrogels in various biomedical applications, particularly in cartilage repair, where the hydrogel must endure mechanical forces from joint movement while simultaneously supporting new tissue growth. In addition to DN structures, tough hydrogels can be reinforced through the incorporation of nanoparticles, nanofibers, or other nanomaterials [70,71]. These reinforcing agents are dispersed throughout the hydrogel matrix, where they interact with the polymer chains to enhance the material’s mechanical properties. For instance, graphene oxide, known for its exceptional mechanical strength and large surface area, has been used to reinforce hydrogels, resulting in materials that not only exhibit toughness but also enhanced electrical conductivity [72,73]. This combination of properties makes tough hydrogels suitable for applications in bioelectronics, where both mechanical resilience and electrical functionality are needed [74,75,76,77]. In addition, Graphene-based hydrogels have also been explored for neural tissue engineering, particularly in constructing three-dimensional scaffolds that mimic spinal cord structures. For instance, rolled graphene oxide foams have been shown to support neural fiber growth through electrical stimulation of stem cells, demonstrating both the mechanical resilience and functional versatility of graphene-enhanced hydrogels [78]. This approach highlights the potential of tough hydrogels to be applied in load-bearing, electrically conductive environments, further expanding their application in tissue regeneration. Graphene oxide has been widely used to reinforce hydrogels due to its exceptional mechanical strength and large surface area, resulting in materials with superior strength and enhanced electrical conductivity. However, in addition to graphene, carbon nanotubes (CNTs) have gained significant attention, particularly in applications requiring high electrical conductivity, such as cardiac tissue engineering. The study “Electrically conductive carbon-based (bio)-nanomaterials for cardiac tissue engineering” demonstrates that CNTs, with their excellent biocompatibility and high conductivity, are well-suited for promoting cellular activity in electrically stimulated tissues, such as the heart [79]. Furthermore, graphene has been successfully applied as a three-dimensional scaffold in various tissue engineering and regenerative medicine applications. Graphene-based 3D scaffolds exhibit a combination of biocompatibility, biodegradability, and mechanical resilience, while maintaining high electrical conductivity [80]. These properties make graphene an ideal platform for complex tissue environments, such as cardiac, neural, and bone tissue regeneration. The combined use of carbon-based nanomaterials like graphene and CNTs highlights their critical role in developing advanced scaffolds for tissue engineering that require both mechanical strength and electrical functionality. Despite progress in developing tough hydrogels, several challenges remain. One significant concern is the potential trade-off between toughness and other desirable properties such as biocompatibility and biodegradability [56,81]. Incorporating rigid synthetic polymers or reinforcing nanomaterials may reduce biocompatibility or make the hydrogels harder to degrade within the body [82]. To overcome these issues, researchers are exploring the use of natural polymers and biodegradable materials in the design of tough hydrogels, as well as integrating bioactive molecules that promote tissue regeneration while maintaining mechanical properties [11,83,84]. The application of nanomaterial-based hydrogels in neural tissue engineering is an emerging and significant area of research. Graphene, in particular, offers unique advantages due to its excellent electrical conductivity and mechanical properties, which are essential for neural regeneration. For instance, the study “Graphene scaffolds in progressive nanotechnology/stem cell-based tissue engineering of the nervous system” highlights how graphene scaffolds play a pivotal role in supporting stem cell-based neural tissue regeneration [85]. The study demonstrates that graphene enhances cell adhesion and provides the necessary electrical cues to promote neural signaling, which is crucial for nerve repair. Furthermore, the study “Hydrogel-integrated graphene superstructures for tissue engineering: From periodontal to neural regeneration” demonstrates the successful integration of graphene and hydrogels in various tissue regeneration applications, including neural tissue [86]. This research illustrates that when graphene is combined with hydrogels, the resulting composite benefits from both the electrical conductivity of graphene and the biocompatibility of the hydrogel, creating an optimal environment for neural tissue repair. These graphene-integrated hydrogel systems can accelerate the repair of damaged neural tissues while facilitating electrical stimulation, which is essential for restoring neural function. Thus, the integration of nanomaterials such as graphene into hydrogels represents a promising approach in neural tissue engineering. These composite systems offer a unique combination of biocompatibility, mechanical strength, and electrical conductivity, making them highly suitable for promoting the regeneration of damaged neural tissues and offering innovative solutions in regenerative medicine. Long-term stability and performance in dynamic biological environments are also ongoing challenges. For load-bearing applications, such as cartilage repair, the hydrogel must withstand repeated mechanical loading and maintain its structural integrity over time [87]. This requires careful optimization of the hydrogel’s composition and structure to balance toughness with other critical factors such as swelling behavior, degradation rate, and interactions with biological tissues [88]. Future research is focused on developing novel hydrogel formulations, advanced fabrication techniques, and comprehensive in vivo testing to evaluate the long-term performance of tough hydrogels in clinical settings.

### 2.3. Smart Hydrogels

Smart hydrogels represent a significant advancement in biomaterials, offering precise control over drug delivery, tissue engineering, and other biomedical applications through their responsiveness to environmental stimuli [89]. These hydrogels are engineered to undergo reversible changes in their physical or chemical properties in response to external factors such as pH, temperature, light, ionic strength, or specific biomolecules [89,90,91]. This adaptive behavior allows smart hydrogels to provide targeted and controlled therapeutic effects, making them highly versatile tools in modern medicine. One of the most extensively studied types of smart hydrogels is pH-sensitive hydrogels. These materials are designed to swell or shrink in response to pH changes, enabling controlled drug release in specific environments [92,93]. For example, in cancer therapy, pH-sensitive hydrogels can deliver chemotherapeutic agents directly to the tumor site where the acidic microenvironment triggers drug release. This targeted approach minimizes the exposure of healthy tissues to toxic chemotherapy, reducing side effects and enhancing treatment efficacy [94,95]. In addition, pH regulation is a critical factor in the gelation process of chitosan hydrogels used as templates for tissue regeneration. For example, the study “pH-sensitive chitosan hydrogel with instant gelation for myocardial regeneration” demonstrates how pH-sensitive chitosan hydrogels can rapidly gelate, supporting their application in myocardial regeneration [96]. This pH-dependent gelation mechanism is instrumental in controlling the physical properties of chitosan hydrogels, making them an effective platform for various tissue regeneration applications. The ability to tailor the hydrogel’s properties in response to pH changes enhances the development of customized biomaterials suited to specific regenerative environments. Temperature-sensitive or thermos-responsive hydrogels are another widely researched category. These hydrogels undergo a sol-gel transition in response to temperature changes, which can be utilized in various biomedical applications [97,98,99]. For instance, thermos-responsive hydrogels can be injected as a liquid at room temperature and then form a gel at body temperature, providing a localized depot for sustained drug release [99,100,101]. This property is particularly useful for treating chronic conditions where long-term drug delivery is needed. Additionally, thermos-responsive hydrogels are being explored for use in minimally invasive surgery, where their ability to conform to tissue shapes can enhance surgical precision and effectiveness [97]. Light-responsive hydrogels, also known as photo-responsive hydrogels, have garnered significant attention due to their ability to respond to light exposure [102]. These hydrogels can be engineered to release drugs, change their mechanical properties, or alter their shape in response to specific wavelengths of light. This capability provides precise spatial and temporal control over the hydrogel’s behavior, making them ideal for applications in tissue engineering and regenerative medicine. For example, light-responsive hydrogels can create dynamic scaffolds that modify stiffness or porosity in response to light, facilitating controlled cell growth and differentiation [103]. Ion-responsive hydrogels are another type of smart hydrogel that reacts to changes in the ionic strength of the surrounding environment [102]. These hydrogels can be designed to release therapeutic agents in response to specific ions such as calcium or potassium, making them useful for applications in bone regeneration or cardiovascular therapy. For instance, calcium-sensitive hydrogels can deliver osteogenic factors in response to elevated calcium levels during bone healing, enhancing the regeneration process [104]. In addition, there are numerous methods for stimulating stem cell differentiation, each of which plays a pivotal role in directing stem cells toward specific lineages. For instance, thermal stimulation has been shown to be effective in promoting stem cell differentiation. The study “The use of graphene in the self-organized differentiation of human neural stem cells into neurons under pulsed laser stimulation” demonstrates how pulsed laser stimulation, utilizing graphene, can facilitate the self-organization and differentiation of neural stem cells into neurons [105]. This research highlights the efficiency of non-contact thermal stimuli in guiding stem cell differentiation. Chemical stimulation is another critical approach, particularly in enhancing electrical signaling within neural networks. The chemical stimuli are shown to augment the electrical communication between neurons cultured on graphene films, thereby improving the functional connectivity of neural cells and facilitating the formation of more robust neural networks [106]. Near-infrared (NIR) laser stimulation also offers promising results, particularly in neural differentiation. The study “Near infrared laser stimulation of human neural stem cells into neurons on graphene nanomesh semiconductors” illustrates how NIR laser stimulation can induce the differentiation of human neural stem cells into neurons when applied to a graphene nanomesh semiconductor platform [107]. NIR stimulation is especially advantageous for deep tissue applications, as it can penetrate biological tissues more effectively and trigger differentiation processes. Electrical stimulation is a well-established method for promoting cell-to-cell interactions, particularly in neural tissues. The study “The control of neural cell-to-cell interactions through non-contact electrical field stimulation using graphene electrodes” highlights how non-contact electrical stimulation through graphene electrodes can regulate neural cell interactions and enhance their functional connectivity, promoting more efficient neural differentiation [108]. Flash photo stimulation is another emerging method, as demonstrated in the study “Flash photo stimulation of human neural stem cells on graphene/TiO_2_ heterojunction for differentiation into neurons” [109]. This research shows how flash photo stimulation on a graphene/TiO_2_ heterojunction platform can rapidly induce neural differentiation, providing a novel and efficient method for triggering stem cell differentiation via light stimuli. Morphological stimulation, in contrast, focuses on the physical environment of the stem cells. The study “Graphene nanogrids for selective and fast osteogenic differentiation of human mesenchymal stem cells” demonstrates how graphene nanogrids can selectively and rapidly promote osteogenic differentiation in human mesenchymal stem cells [110]. This method highlights the importance of controlling the physical microenvironment in guiding stem cell behavior and differentiation. These various stimulation methods, including thermal, chemical, NIR laser, electrical, flash photo, and morphological stimuli, all play critical roles in promoting stem cell differentiation. The selection and application of these stimuli can be tailored to specific regenerative medicine and tissue engineering goals, providing innovative approaches for enhancing stem cell-based therapies. While smart hydrogels offer remarkable potential, their complexity presents challenges in design, fabrication, and clinical translation [111]. Ensuring the stability, reproducibility, and safety of smart hydrogels in real-world applications requires a deep understanding of their interactions with biological environments as well as advanced manufacturing techniques for large-scale production [1]. Future research will likely focus on expanding the range of stimuli that smart hydrogels can respond to and enhancing their sensitivity to existing stimuli. The integration of smart hydrogels with technologies such as nanotechnology, bioelectronics, and gene therapy promises to revolutionize next-generation biomedical devices and treatments, delivering highly targeted therapies with minimal side effects [112].

### 2.4. Hybrid Hydrogels

Hybrid hydrogels combine the advantages of natural and synthetic polymers, creating materials that exhibit both the biocompatibility and bioactivity of natural polymers and the tunable mechanical properties and stability of synthetic ones. These materials are designed to overcome the limitations of conventional hydrogels, particularly in applications where a single type of polymer is insufficient to meet the mechanical, chemical, and biological demands. Hybrid hydrogels have opened new avenues in tissue engineering, drug delivery, and regenerative medicine where complex environments and diverse functional requirements necessitate multifunctional materials [113]. One of the key benefits of hybrid hydrogels is their ability to incorporate multiple functionalities within a single material [114]. For example, a hybrid hydrogel may combine the elasticity and cell-adhesive properties of a natural polymer such as collagen or hyaluronic acid with the mechanical strength and degradability of a synthetic polymer like poly(lactic-co-glycolic acid) (PLGA). This combination allows the hydrogel to provide structural support for tissue regeneration while also offering biological cues for cell proliferation and differentiation [115]. In tissue engineering, this leads to scaffolds that support tissue growth while actively guiding the development of specific cell types or tissue structures. Beyond blending polymers, hybrid hydrogels can also incorporate nanoparticles, nanofibers, or other nanomaterials to enhance their properties [116,117]. For instance, embedding gold nanoparticles in a hydrogel matrix can confer photo-thermal properties, enabling the material to be used in cancer therapy where localized heating can ablate tumor cells [118,119]. Similarly, the inclusion of magnetic nanoparticles allows for the creation of magnetically responsive hydrogels which can be manipulated using external magnetic fields for targeted drug delivery or controlled therapeutic agent movement within the body [120]. Hybrid hydrogels also offer an ideal platform for the controlled delivery of multiple therapeutic agents [114,121,122]. By integrating polymers with different degradation rates, hybrid hydrogels can be engineered to release drugs, proteins, or nucleic acids in a sequential or sustained manner, enabling sophisticated drug delivery approaches. For example, in wound healing, a hybrid hydrogel could first release an antimicrobial agent to prevent infection followed by the release of growth factors to promote tissue regeneration as healing progresses [123,124,125]. This controlled release timing and sequence are particularly valuable in treating complex conditions that require different therapies at various stages of healing. Another promising application of hybrid hydrogels lies in regenerative medicine where these materials can be used to create bioactive scaffolds that mimic the natural extracellular matrix (ECM) of tissues. By incorporating ECM components like collagen, laminin, or fibronectin into the hydrogel matrix, researchers can design scaffolds that promote cell attachment, migration, and differentiation, essential for regenerating difficult-to-repair tissues such as cartilage, bone, and nerves [126,127]. Moreover, by adjusting the mechanical properties and degradation rates of the synthetic components, hybrid hydrogels can be tailored to the specific needs of different tissues, providing a more personalized approach to tissue engineering [128]. Despite their many advantages, hybrid hydrogels present challenges in synthesis, characterization, and application. One difficulty lies in ensuring the uniform distribution of components and consistent behavior of the material under physiological conditions. Additionally, the potential for immune responses to the natural components of hybrid hydrogels must be carefully considered, especially in long-term applications where the material will interact with the immune system [114,129]. Researchers are addressing these challenges through innovative fabrication techniques such as microfluidics, electrospinning, and 3D printing, which offer precise control over the composition and structure of hybrid hydrogels. Looking ahead, the future of hybrid hydrogels is promising, with ongoing research aimed at expanding their functionality and exploring new applications. The integration of hybrid hydrogels with cutting-edge technologies such as stem cell therapy, gene editing, and bioelectronics holds immense potential for developing next-generation therapies that address critical medical challenges [130]. As our understanding of the interactions between different polymers, nanoparticles, and biological molecules deepens, hybrid hydrogels are expected to play a pivotal role in advancing innovative treatments for a wide range of diseases and conditions.

## 3. Hydrogel Applications

### 3.1. Skin Regeneration

Skin serves as a vital barrier against environmental threats while maintaining homeostasis. However, due to its constant exposure to external factors, it is prone to injuries such as cuts, burns, and chronic wounds [131,132]. The complex structure of skin, with its multiple layers and diverse cell types, presents significant challenges for effective regeneration after damage. Hydrogels have emerged as a promising solution, offering a moist environment that not only accelerates healing but also lowers the risk of infection and encourages tissue formation [133,134]. Previous wound dressings provide protection but often fail to create the optimal conditions for healing [135]. In contrast, hydrogels can be designed to deliver bioactive molecules such as growth factors, cytokines, and antimicrobial agents directly to the wound, enhancing the body’s natural healing processes [18,136,137]. For instance, hydrogels infused with vascular endothelial growth factor (VEGF) can promote the formation of new blood vessels, critical for delivering oxygen and nutrients to regenerating tissue [138,139]. Incorporating antimicrobial peptides can further prevent infections by targeting specific pathogens while safeguarding healthy tissue. Advanced hydrogels, which respond to environmental changes, have brought further innovations in wound care [46,140]. These materials can release therapeutic agents based on the wound’s condition, such as changes in pH or temperature. For example, a pH-sensitive hydrogel may release antibiotics when the wound environment becomes more acidic, indicating infection, thereby reducing unnecessary exposure to drugs and minimizing the risk of antibiotic resistance [141,142]. Similarly, temperature-sensitive hydrogels adjust their properties in response to body heat, creating a more adhesive and supportive dressing as healing progresses [143]. Injectable hydrogels represent another advancement, particularly for treating deep or irregular wounds that are difficult to cover with standard dressings [46]. These hydrogels are administered directly into the wound and quickly form a gel that conforms to its shape, providing a matrix that supports cell infiltration and tissue regeneration. By incorporating stem cells or other regenerative elements, injectable hydrogels can enhance the healing process, promoting the regeneration of skin and minimizing scarring [144]. Despite the advancements in hydrogel-based treatments, several challenges remain. Ensuring the long-term stability and biocompatibility of these materials, especially in chronic wounds requiring prolonged treatment, remains a key concern [48,145]. Additionally, the possibility of hydrogels triggering immune responses or breaking down into harmful by-products needs careful consideration, particularly for patients with weakened immune systems [9]. Ongoing research is focused on developing hydrogels that are both effective and safe for extended use [146]. In the future, integrating hydrogels with advanced wound care technologies like negative pressure wound therapy (NPWT) and bioactive dressings holds great potential for improving the treatment of complex wounds. Furthermore, the creation of hydrogels that can release multiple therapeutic agents in a controlled manner, tailored to the specific healing stages of the wound, represents a promising research direction [4]. As our understanding of wound healing biology continues to expand, hydrogels are likely to become increasingly central to the development of more effective skin injury treatments.

### 3.2. Cartilage Regeneration

Cartilage is a specialized connective tissue crucial for joint function, providing a smooth lubricated surface for movement and enabling load transmission with minimal friction [147]. However, its avascular nature and limited regenerative capacity make it highly susceptible to damage from injuries or degenerative conditions like osteoarthritis [148,149,150]. Cartilage regeneration poses a significant challenge in tissue engineering, as materials must not only support cell growth and differentiation but also replicate the mechanical properties of natural cartilage [148,151,152]. Hydrogels have shown great potential in cartilage regeneration due to their ability to provide a three-dimensional environment conducive to the growth of chondrocytes, the cells responsible for cartilage formation [153,154]. Tough hydrogels are particularly suitable for this purpose, as they can withstand the mechanical forces exerted by surrounding joints while fostering tissue regeneration [56,70,104]. These hydrogels can be designed to mimic the structure and mechanical features of natural cartilage, serving as a scaffold for new tissue growth [155]. A key approach in hydrogel-based cartilage regeneration involves integrating bioactive molecules such as growth factors and signaling peptides to stimulate chondrogenesis [104,156,157]. For example, hydrogels containing transforming growth factor-beta (TGF-β) have been shown to promote the differentiation of mesenchymal stem cells (MSCs) into chondrocytes, resulting in new cartilage formation [158,159]. Incorporating extracellular matrix components like hyaluronic acid and chondroitin sulfate into the hydrogel matrix can further enhance its chondrogenic potential by providing biochemical signals that replicate the native cartilage environment [160,161]. Smart hydrogels take cartilage regeneration a step further by responding to mechanical or biochemical cues within the joint [104,162]. These hydrogels can be engineered to release growth factors or other therapeutic agents in response to mechanical stress or pH changes, offering a dynamic and adaptive treatment [1]. For instance, a hydrogel designed to release TGF-β in response to compressive forces could accelerate the regeneration of load-bearing cartilage without promoting overgrowth in areas experiencing less stress. Injectable hydrogels are also being developed for minimally invasive cartilage repair [154,163]. These hydrogels can be delivered directly into cartilage defects, where they form a gel that conforms to the defect’s shape and provides a scaffold for tissue regeneration. Injectable hydrogels can be loaded with stem cells, growth factors, or bioactive molecules, making them a versatile tool for cartilage repair. For example, injectable hydrogels containing MSCs have demonstrated enhanced cartilage repair in animal studies by promoting the formation of new cartilage and reducing inflammation [164,165]. Despite the promising outcomes of hydrogel-based cartilage therapies, several challenges persist. A major concern is the long-term stability and durability of the hydrogel scaffold, especially in the dynamic environment of a joint [166,167]. The hydrogel must endure repeated mechanical loading without losing its structure or degrading prematurely [60]. To address this, researchers are exploring hybrid hydrogels that combine the toughness of synthetic polymers with the biocompatibility of natural materials, as well as the addition of reinforcing agents such as nanofibers or nanoparticles to improve the mechanical strength of the scaffold [168,169]. Another challenge is ensuring that the newly formed cartilage integrates seamlessly with surrounding native tissue [147,151,170]. This requires carefully promoting chondrogenesis while controlling the inflammatory response and avoiding fibrosis, which could impede proper tissue integration. Furthermore, scalability remains an issue, as producing and characterizing these materials is often complex and time-consuming. Future research will likely focus on overcoming these challenges by developing novel hydrogel formulations and integrating hydrogel-based therapies with other regenerative strategies such as gene therapy and advanced tissue engineering techniques [171,172].

### 3.3. Drug Delivery

**Figure 2:**
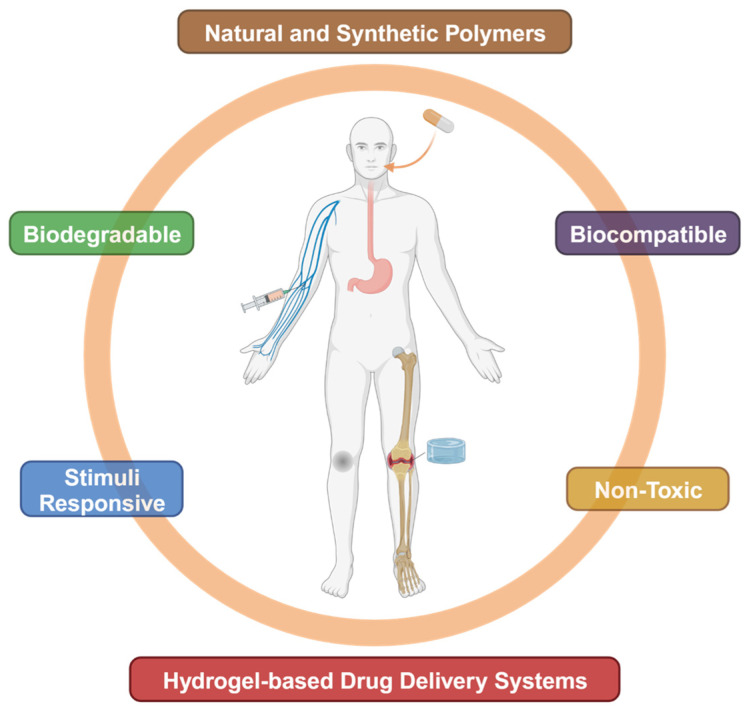
Various types of hydrogel for drug delivery.

The development of advanced drug delivery systems (Figure 2) has become a key aspect of modern medicine, allowing targeted delivery of therapeutic agents to specific tissues, maximizing efficacy while minimizing side effects [122,173]. Hydrogels, with their ability to encapsulate and release a wide range of drugs, have emerged as a versatile platform for drug delivery [174]. These materials can be designed to control the release of drugs over time, respond to environmental stimuli, or deliver drugs in a targeted manner, making them ideal for treating chronic diseases, localized conditions, and complex multi-drug therapies [6]. One major benefit of hydrogels in drug delivery is their capacity to provide sustained release of therapeutic agents [175]. previous drug delivery methods often cause rapid drug release, leading to a sharp increase in drug concentration followed by a rapid decline [176,177]. In contrast, hydrogels can be tailored to release drugs gradually over an extended period, maintaining therapeutic levels and reducing the frequency of administration [4,7]. This is especially valuable for chronic conditions such as diabetes or cancer, where continuous drug delivery is crucial for effective disease management [178,179]. Smart hydrogels take drug delivery further by introducing responsiveness to environmental factors such as changes in pH, temperature, or specific ions or biomolecules [112]. This allows for on-demand drug release, ensuring the drug is delivered only when necessary, reducing side effects, and improving patient adherence [180]. For example, pH-sensitive hydrogels can deliver anticancer drugs directly to the acidic environment of tumors, ensuring that the drug is released only at the target site [95]. Similarly, thermos-responsive hydrogels can be injected as a liquid and form a gel depot at body temperature, providing sustained drug release at the site of injection [97,181,182]. In addition to systemic drug delivery, hydrogels are being explored for localized treatments [183,184,185]. Injectable hydrogels, for instance, can deliver drugs directly to specific tissues or organs, minimizing systemic exposure and reducing adverse effects [186,187]. These hydrogels can be loaded with various therapeutic agents, including small molecules, proteins, peptides, and nucleic acids, making them highly versatile for treating multiple diseases. For example, injectable hydrogels loaded with chemotherapeutic agents have been developed for localized tumor treatment, where the hydrogel forms a depot at the tumor site, slowly releasing the drug to maximize its therapeutic effect [188,189]. Hybrid hydrogels, which combine natural and synthetic polymers, offer a more sophisticated approach to drug delivery. Chitosan has emerged as a highly suitable natural polymer for the development of hydrogels used in tissue engineering and drug delivery, owing to its favorable biocompatibility and biodegradability. Recent advancements, such as those presented in “Customizing nano-chitosan for sustainable drug delivery”, highlight the potential of nano-chitosan in creating sustainable and efficient drug delivery systems [190]. Additionally, the comprehensive review “A review on chitosan-based biomaterial as carrier in tissue engineering and medical applications” emphasizes the broad utility of chitosan in tissue engineering [191]. Furthermore, research such as “Prevascularized micro-/nano-sized spheroid/bead aggregates for vascular tissue engineering” illustrates the application of micro/nano-scale polymers in tissue regeneration, underscoring the versatility of these materials [192]. Collectively, these studies showcase the promising role of chitosan and related polymer systems in advancing the fields of drug delivery and tissue regeneration. These hydrogels can be engineered to respond to multiple stimuli such as pH and temperature, allowing for precise and controlled drug release tailored to the patient’s specific needs. Hybrid hydrogels can also encapsulate multiple therapeutic agents, enabling combination therapies where two or more drugs are released simultaneously or sequentially in response to different triggers [7,121,175,193]. Despite the advances in hydrogel-based drug delivery systems, several challenges remain. A primary concern is the risk of drug degradation or denaturation within the hydrogel matrix, particularly for sensitive biological molecules like proteins or peptides [122,194]. Researchers are exploring protective coatings or encapsulation techniques to stabilize the drug within the hydrogel and prevent degradation. Ensuring the biocompatibility and safety of hydrogels, especially for long-term drug delivery, presents another challenge. This requires a thorough understanding of the interactions between the hydrogel and surrounding tissues, as well as the potential for immune responses or other adverse effects [2,3]. Future research in hydrogel-based drug delivery systems will likely focus on overcoming these challenges and exploring new applications for these versatile materials. For instance, integrating hydrogels with nanotechnology could lead to the development of smart drug delivery systems capable of targeting specific cells or tissues with remarkable precision [4,112,122,195]. Additionally, combining hydrogels with other advanced materials such as bioelectronics or gene therapy could open new doors for treating a wide range of diseases [196].

### 3.4. Expanded Discussion on Hydrogel Applications

Hydrogels have become a cornerstone of modern biomedical science due to their unique physicochemical properties, which make them highly suitable for a wide range of applications, including tissue regeneration, drug delivery, and medical implants. To provide a more comprehensive understanding of their use, we will explore key case studies across several fields (Table 2): 1. Skin Regeneration: One of the primary applications of hydrogels is in wound healing and skin regeneration. A notable case study involves the use of collagen-based hydrogels that promote re-epithelialization and wound closure in burn patients. Studies such as Boateng et al. demonstrated that these hydrogels not only accelerate wound healing but also support cellular proliferation, leading to faster recovery times in severe wounds [197]. The development of hydrogels infused with growth factors, such as epidermal growth factor (EGF), further enhances skin regeneration by stimulating keratinocyte migration. 2. Cartilage Repair: In the field of orthopedic tissue engineering, synthetic hydrogels such as PEG-based systems have shown great promise in cartilage repair. These hydrogels mimic the native extracellular matrix (ECM) of cartilage and provide the mechanical support necessary for chondrocyte activity. A significant case study by Elisseeff et al. demonstrated the use of hybrid hydrogels that support both the mechanical strength and biological function of cartilage tissues [198]. However, challenges remain in mimicking the complex zonal architecture of native cartilage, an area where further research is actively being conducted. 3. Drug Delivery: Smart hydrogels capable of controlled drug release have become a significant focus in pharmaceutical research. For instance, Buwalda et al. explored the application of pH-sensitive hydrogels for the targeted delivery of anticancer drugs, releasing the drug only in the acidic tumor environment. Similarly, temperature-responsive hydrogels have been used to deliver chemotherapy drugs to specific sites in the body, providing on-demand release and minimizing systemic side effects [23]. These innovations underscore the potential of hydrogels to revolutionize the field of precision medicine. 4. Tissue Engineering: Conductive hydrogels have been explored for their ability to regenerate neural tissues by enhancing neural signaling pathways. Studies such as Liu et al. focused on graphene-based hydrogels that provide both structural support and electrical conductivity to promote neural repair [24]. These hydrogels are critical in spinal cord injury treatments, where the regeneration of neural networks is vital for recovery. Additionally, hydrogels loaded with bioactive molecules, such as neurotrophic factors, have shown promise in enhancing nerve growth and repair. 5. Bone Regeneration: In the realm of bone tissue engineering, nano-hydroxyapatite-incorporated hydrogels have been shown to promote osteogenesis and support the regeneration of bone tissues. A key case study by Lee and Mooney demonstrated how these hydrogels not only support the growth of osteoblasts but also improve the mechanical properties of the scaffold, facilitating better integration with the host bone tissue [199]. This approach has significant implications for the treatment of bone defects and fractures. In addition, In recent years, sericin-based hydrogels have attracted significant attention due to their exceptional physicochemical properties and biological activity, making them promising materials in tissue engineering and regenerative medicine. Sericin, a natural protein, is particularly effective in promoting the repair and regeneration of damaged tissues, including skin, muscle, and nerve tissues. Due to its high biocompatibility and biodegradability, sericin-based hydrogels are widely used in skin regeneration and wound healing, and they are emerging as a promising material for muscle and nerve tissue repair. One of the key advantages of sericin is its strong moisture retention capability, which is critical for wound healing and skin regeneration. Furthermore, sericin exhibits anti-inflammatory and antimicrobial properties, helping to reduce the risk of infection and accelerating the healing of damaged tissues. In muscle and nerve tissue repair, the bioactivity of sericin supports cellular interactions and promotes the regeneration of damaged neural pathways. Recent studies have demonstrated that sericin-based hydrogels are not only effective in skin applications but also hold great potential for broader tissue regeneration uses. For instance, hydrogels incorporating sericin have been shown to significantly enhance wound healing rates and improve skin restoration following surgical procedures. In muscle and nerve regeneration, research has validated sericin’s outstanding biocompatibility and regenerative effects, further expanding its potential as a valuable biomaterial in these areas. Incorporating the latest findings on sericin-based hydrogels into this review will help readers gain a comprehensive understanding of the current advancements in hydrogel research. By highlighting the expanding applications of sericin-based hydrogels, this discussion aims to provide deeper insights into the development and utilization of biomaterials in tissue regeneration.

## 4. Recent Advancements and State-of-the-Art in Hydrogel Technology

In recent years, hydrogels have garnered significant attention as versatile materials with broad applications in tissue engineering, drug delivery, and regenerative medicine. The continuous advancement of hydrogel technology has led to the development of more sophisticated systems that offer improved mechanical properties, biocompatibility, and functional versatility. This chapter provides a comprehensive overview of the most recent advancements in hydrogel research, with a focus on state-of-the-art innovations in smart hydrogels, hybrid systems, and their emerging biomedical applications. One of the most remarkable advancements in hydrogel technology is the development of smart hydrogels, which are capable of responding to various environmental stimuli, such as pH, temperature, or specific biomolecules. These stimuli-responsive hydrogels have paved the way for more efficient and targeted drug delivery systems. For instance, pH-sensitive hydrogels can be designed to release drugs only in specific microenvironments, such as the acidic conditions typically found in tumor tissues. Research by Yan et al. demonstrated that pH-sensitive hydrogels could precisely deliver anticancer agents to tumor sites, minimizing off-target effects and improving therapeutic outcomes [202]. Similarly, thermo-responsive hydrogels have been developed for localized drug delivery. These hydrogels remain in a liquid state at lower temperatures but form a solid gel at physiological temperatures, allowing them to be easily injected into the body and form a depot at the target site. Studies like Santhamoorthy et al. have highlighted the potential of thermo-responsive hydrogels in delivering drugs over an extended period, particularly in the context of chronic disease management where sustained drug release is crucial [203]. Smart hydrogels have also been designed to respond to specific biomolecules, enabling on-demand drug release in response to physiological changes. This capability not only enhances the precision of drug delivery but also reduces side effects by ensuring that therapeutic agents are released only when needed. The integration of biomolecule-sensitive hydrogels has shown great promise in treating diseases like diabetes, where hydrogels can release insulin in response to glucose levels [204]. The development of hybrid hydrogels, which combine both natural and synthetic polymers, represents another significant advancement in the field. These systems leverage the biocompatibility and biodegradability of natural polymers while incorporating the mechanical strength and tunability of synthetic materials. Hybrid hydrogels have been particularly effective in enhancing drug delivery systems and tissue engineering scaffolds due to their ability to respond to multiple stimuli and their capacity to encapsulate multiple therapeutic agents. For example, research by Hoare et al. demonstrated that hybrid hydrogels could be engineered to release drugs in a sequential manner, responding to changes in pH and temperature [205]. This dual-stimuli responsiveness allows for precision in drug release, particularly in cases where combination therapies are required. In tissue engineering, hybrid hydrogels have been used to provide a supportive matrix for cell growth, differentiation, and tissue regeneration. The mechanical properties of these materials can be fine-tuned to mimic the natural extracellular matrix (ECM) of specific tissues, promoting better integration and functionality. Hydrogels have been extensively studied as scaffolds in tissue engineering, particularly for the regeneration of complex tissues such as neural and muscle tissues. Recent advancements have focused on improving the structural integrity, electrical conductivity, and bioactivity of hydrogels to better support tissue regeneration. In neural tissue engineering, conductive hydrogels have emerged as a promising material for promoting neural repair. Studies like Green et al. have shown that incorporating conductive elements such as graphene or carbon nanotubes into hydrogel matrices enhances cell signaling and promotes the growth and differentiation of neural cells [206]. These conductive hydrogels provide a platform for guiding nerve regeneration and restoring damaged neural pathways. Similarly, in muscle tissue engineering, hydrogels have been developed to support the regeneration of damaged muscle fibers. Hydrogels that incorporate bioactive molecules such as growth factors can accelerate muscle repair by promoting cellular proliferation and differentiation [207]. Additionally, the mechanical properties of hydrogels can be adjusted to mimic the elasticity of muscle tissue, providing a supportive environment for muscle regeneration. Despite the remarkable advancements in hydrogel technology, several challenges remain. A major concern is the stability of drugs encapsulated within hydrogels, particularly for sensitive biological molecules such as proteins or peptides. Researchers are actively exploring strategies such as protective coatings or encapsulation techniques to prevent degradation and ensure the sustained release of bioactive agents. Ensuring the long-term biocompatibility and safety of hydrogels is another critical area of research. While many hydrogels are designed to be biodegradable, their interactions with surrounding tissues, as well as potential immune responses, must be thoroughly evaluated to prevent adverse effects, especially in long-term applications. Looking forward, the integration of hydrogels with nanotechnology and bioelectronics presents exciting opportunities for creating next-generation drug delivery systems and tissue scaffolds. By combining hydrogels with advanced materials, it may become possible to develop intelligent systems capable of targeting specific cells or tissues with unparalleled precision. Additionally, innovations such as the use of gene therapy or bioelectronic interfaces in combination with hydrogels could unlock new possibilities for treating a wide range of diseases.

## 5. Conclusions

Advancements in hydrogel technology represent a major breakthrough in biomaterials, providing innovative solutions to key challenges in modern medicine. Self-healing, tough, smart, and hybrid hydrogels have opened new avenues in tissue engineering, drug delivery, and regenerative medicine. Self-healing hydrogels, capable of autonomous repair, offer the potential for longer-lasting implants and more durable drug delivery systems, although balancing this feature with mechanical strength remains a challenge. Tough hydrogels have extended applications in fields like cartilage repair, but ensuring long-term biocompatibility and stability is essential. Smart hydrogels, which respond to environmental stimuli, allow for precise control over drug delivery, and ongoing research aims to further enhance their role in personalized medicine. Hybrid hydrogels, which combine natural and synthetic polymers, provide versatile platforms for complex biomedical applications, though synthesis challenges remain. As research into hydrogels continues, these materials are expected to play an increasingly important role in next-generation medical devices and therapies. Continued innovation and interdisciplinary collaboration will be crucial for translating laboratory successes into clinical applications, with the potential to revolutionize the treatment of various diseases and greatly improve patient outcomes.

## Figures and Tables

**Table 1:** Comparison of hydrogel types: properties and applications.

Hydrogel Type	Key Properties	Applications
Self-Healing Hydrogels [19,20]	Dynamic covalent bonds	Tissue engineering
	Self-repair capabilities	Skin regeneration
	Suitable for repeated use	Cartilage regeneration
Tough Hydrogels [21,22]	High mechanical strength	Cartilage regeneration
	Double network structures	Artificial ligaments
	Improved durability	Biomedical implants
Smart Hydrogels [23,24]	Environment-responsive	Drug delivery
	Controlled drug release	Precision medicine
		Personalized therapies
Hybrid Hydrogels [25]	Combination of natural	Complex tissue engineering
	Synthetic polymers	Regenerative medicine
	Tunable mechanical properties	Personalized therapies

**Table 2:** Comparison of Hydrogel Applications in Skin Regeneration, Cartilage Repair, and Drug Delivery.

Applications	Hydrogel Type	Key Features	Advantages	Challenges
Skin Regeneration [197,200]	Natural polymer hydrogels (e.g., collagen, hyaluronic acid)	Biocompatibility Promotes	Accelerates re-epithelialization	Limited mechanical strength
		Wound healing	Supports cell proliferation	Degradation control
Cartilage Regeneration [198,201]	Synthetic hydrogels (e.g., PEG, PVA)	High mechanical strength	Mimics native cartilage properties	Difficulty in mimicking complex cartilage structure
	Hybrid hydrogels	Load-bearing	Supports chondrocyte activity	Slow integration
Drug Delivery [23,24]	Smart hydrogels (e.g., pH and temperature sensitive)	Responsive to stimuli	Precise control over drug release profiles	Limited long-term stability
		Controlled release	Reduces side effects	Potential immune response

## Data Availability

No new data were created or analyzed in this study. Data sharing is not applicable to this article.
